# Integrated Analysis of mRNA and MicroRNA Co-expressed Network for the Differentiation of Bovine Skeletal Muscle Cells After Polyphenol Resveratrol Treatment

**DOI:** 10.3389/fvets.2021.777477

**Published:** 2021-12-24

**Authors:** Dan Hao, Xiao Wang, Yu Yang, Bo Thomsen, Lars-Erik Holm, Kaixing Qu, Bizhi Huang, Hong Chen

**Affiliations:** ^1^College of Animal Science and Technology, Northwest A&F University, Shaanxi Key Laboratory of Animal Genetics, Breeding and Reproduction of Shaanxi Province, Yangling, China; ^2^Department of Molecular Biology and Genetics, Aarhus University, Aarhus, Denmark; ^3^Konge Larsen ApS, Kongens Lyngby, Denmark; ^4^Academy of Science and Technology, Chuxiong Normal University, Chuxiong, China; ^5^Yunnan Academy of Grassland and Animal Science, Kunming, China; ^6^College of Animal Science, Xinjiang Agricultural University, Urumqi, China

**Keywords:** primary bovine myoblast, resveratrol, differentially expressed analysis, WGCNA, mRNA co-expression, miRNA co-expression, mRNA-miRNA network

## Abstract

Resveratrol (RSV) has been confirmed to benefit human health. Resveratrol supplemented in the feeds of animals improved pork, chicken, and duck meat qualities. In this study, we identified differentially expressed (DE) messenger RNAs (mRNAs) (*n* = 3,856) and microRNAs (miRNAs) (*n* = 93) for the weighted gene co-expression network analysis (WGCNA) to investigate the co-expressed DE mRNAs and DE miRNAs in the primary bovine myoblasts after RSV treatment. The mRNA results indicated that RSV treatments had high correlations with turquoise module (0.91, *P*-value = 0.01) and blue module (0.93, *P*-value < 0.01), while only the turquoise module (0.96, *P*-value < 0.01) was highly correlated with the treatment status using miRNA data. After biological enrichment analysis, the 2,579 DE genes in the turquoise module were significantly enriched in the Gene Ontology (GO) terms and Kyoto Encyclopedia of Genes and Genomes (KEGG) pathways. The top two GO terms were actin filament-based process (GO:0030029) and actin cytoskeleton organization (GO:0030036). The top two KEGG pathways were regulation of actin cytoskeleton (bta04810) and tight junction (bta04530). Then, we constructed the DE mRNA co-expression and DE miRNA co-expression networks in the turquoise module and the mRNA–miRNA targeting networks based on their co-expressions in the key module. In summary, the RSV-induced miRNAs participated in the co-expression networks that could affect mRNA expressions to regulate the primary myoblast differentiation. Our study provided a better understanding of the roles of RSV in inducing miRNA and of the characteristics of DE miRNAs in the key co-expressed module in regulation of mRNAs and revealed new candidate regulatory miRNAs and genes for the beef quality traits.

## Introduction

Resveratrol (RSV) is a natural polyphenol compound found in grapes, nuts, and some blackberries. Researchers have studied its health-promoting effects of neuroprotection (1) and cardioprotection (2) as well as its inhibiting actions to tumor cell proliferation (3) and microbial activity (4) and its diminishing effects on inflammation in humans and animals (5, 6). Its pro-differentiation properties to human lung fibroblasts (7), embryonic cardiomyoblasts (8), and skeletal myoblast have also been studied (9). For example, Dirks Naylor (10) demonstrated the RSV effects on skeletal muscle metabolism, protein catabolism, and muscle-related ischemia and reperfusion injury disease in a review study (10). Resveratrol could also help to improve muscle fatigue resistance (11), reduce aging-induced muscle loss (12), improve muscle atrophy (13), and enhance exercise performance (14). Resveratrol is contained in the wine grape pomace, and adding it to feed will benefit the feed efficiency and meat tenderness in lamb (15). In addition, the antioxidant effects of RSV improved the heat-stressed and the transport-stressed meat quality of broilers (16, 17). Resveratrol alleviated the skeletal muscle mitochondrial dysfunction and oxidative damage, when 80 mg/kg/day RSV was supplied in the intrauterine growth retardation piglets (18). The same dose of RSV also improved the meat quality by increasing the content of oxidative muscle fiber and decreasing the lipid accumulation in pigs (19). Dietary RSV supplements of 300–450 mg/kg in Peking ducks improved meat quality through decreasing abdominal fat rate and shear force, as well-increasing the flavor amino acid and intramuscular fat deposition (20). In cattle, the beneficial effects of RSV have been concluded in several studies on bovine oocyte maturation and subsequent embryonic development (21), inhibition of apoptosis and lipid peroxidation for the fertilization capacity of bovine sex-sorted semen (22), rumen fermentation, methane production, and prokaryotic community composition (23). However, the effect of RSV on beef production and quality still needs further investigation.

The carcass composition of beef cattle is influenced by intrinsic factors (e.g., genetic, age, and sex) and extrinsic factors (e.g., nutrition, environment, and management) (24). Bassel et al. (25) suggested the establishment of global co-expression network connections between genes by considering all samples in *Arabidopsis*. Gene co-expression networks are constructed by genes with significant co-expression relationships, where the co-expressed genes show similar expression patterns across samples that are controlled by the same transcriptional regulatory programs (26, 27). The weighted gene co-expression network analysis (WGCNA) has been used in analyzing the feed efficiency, residual feed intake, carcass traits, and lactation in cattle (28–30).

Our previous study focused on transcriptomic changes in bovine skeletal muscle cells after RSV treatment and was conducted to identify the differentially expressed (DE) genes and microRNAs (miRNAs) (31, 32); therefore, this study mainly focuses on the combined co-expressed transcriptomes, i.e., messenger RNA (mRNA) and miRNA studies for bovine muscle in response to treatment with RSV, which aims to investigate the roles of RSV in inducing miRNA for the better understanding, to identify the characteristics of DE miRNAs in the key co-expressed module in regulation of mRNAs, and to reveal new candidate regulatory miRNAs and genes underlying the beef quality traits.

## Materials and Methods

### Primary Bovine Myoblast, Transcriptome Sequencing, and Differential Expression Analysis

The cultured primary myoblasts from the fetal beef *longissimus dorsi* muscle, the transcriptome sequencing datasets after quality control and alignment, and the differential expression analysis results were achieved from our previous studies (31, 32).

All the animal procedures were carried out according to the protocols approved by the Institutional Animal Care and Use Committee (IACUC) of the College of Animal Science and Technology, Northwest A&F University, China. Ninety-day-old fetal cattle were collected from Tumen slaughterhouse in Xi'an, Shaanxi Province. First, we used 75% alcohol and 1% double-antibody sterile phosphate buffer solution (phosphate buffered saline, PBS) to gently wash the epidermis of the fetal cow to eliminate blood stains and bacterial contamination in an extra-large plate. Second, we cut the muscle tissue pieces and placed them in a 50-ml centrifuge tube, with collagenase I digestion in Dulbecco's modified Eagle's medium (DMEM) at 37°C for 1.5 h. Third, the suspension was filtered and centrifuged, and the supernatant was removed. Fourth, we added four times the volume of 0.25% trypsin in the sediment at 37°C for 30 min. Fifth, the digested sample was filtered through 1-mm stainless steel mesh and 100-μm mesh. Sixth, we added 500 μl of a medium containing 15% fetal bovine serum (FBS) to the pellet to terminate the digestion, and the cells were inoculated in a 6-cm Petri dish. After 20-min culturing, we drew the upper culture medium and continued the culturing process in a new 6-cm Petri dish. When the cell density reached 80–90%, we used them for the subsequent experiments.

RNA for each cell sample was isolated for mRNA sequencing to generate 125 or 150-bp paired-end reads and for miRNA sequencing to generate 50-bp single-end reads on an Illumina Hiseq platform (Illumina, USA). We removed the unqualified reads by quality control to achieve clean reads by in-house *perl* scripts that were used in our previous studies (31, 32). Then, the miRNA tags were mapped to the reference genome of *Bos taurus* (UMD_3.1.1/bosTau8) by Bowtie software (version 0.12.9) (33). The mapped miRNA tags were used to seek the known miRNAs using miRBase20.0 as the reference, so the potential miRNA and the secondary structures were obtained by miRDeep2 software (version 2.0.0.5) (34).

The expected number of fragments per kilobase of transcript sequence per million base pairs sequenced (FPKM) of each gene was calculated to achieve the average FPKM values for the replicates. The threshold of gene expression was set for mRNA, when the FPKM value is larger than 1. Following the normalization formula (35), miRNA expression levels were also estimated by transcript per million (TPM). A differential expression analysis of two groups (case and control) for both mRNA and miRNA was performed using the R package DESeq (version 1.18.0) (36). The *P*-values were adjusted using the Benjamini and Hochberg's method for controlling the false discovery rate (FDR). Differentially expressed mRNAs and DE miRNAs were defined when the adjusted *P*-values were < 0.05. In addition, we calculated fold changes (FCs) between the case and control groups based on the averaged FPKM values and TPM values to define the up-regulated (log_2_FC > 0) and down-regulated (log_2_FC < 0) mRNAs and miRNAs, respectively.

**Figure 1:**
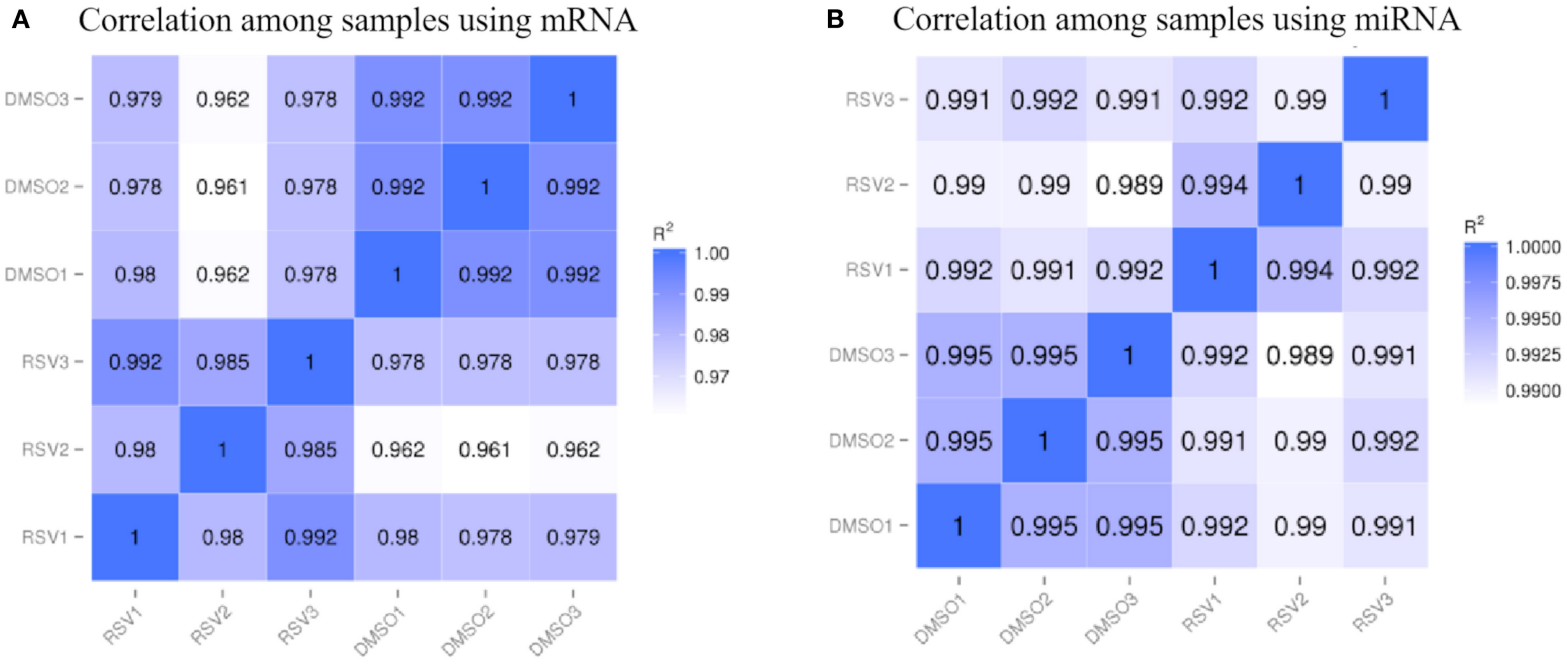
The heatmap of correlation coefficients among samples for **(A)** mRNA data and **(B)** miRNA data. *R*^2^ indicates the square of Pearson correlation coefficient.

We used the skeletal muscle cells under the polyphenol RSV treatment as the case group, while the skeletal muscle cells without RSV treatment were considered as the control group. Meanwhile, both the case and control groups had three independent experiments separately for the skeletal muscle cell collections. The correlation coefficients among samples were visualized in the heatmaps using log_10_(FPKM + 1) for mRNA and log_10_(TPM + 1) for miRNA in Figure 1.

### Gene Co-expression Network of mRNA and miRNA and Their Associations With RSV Treatment

**Figure 2:**
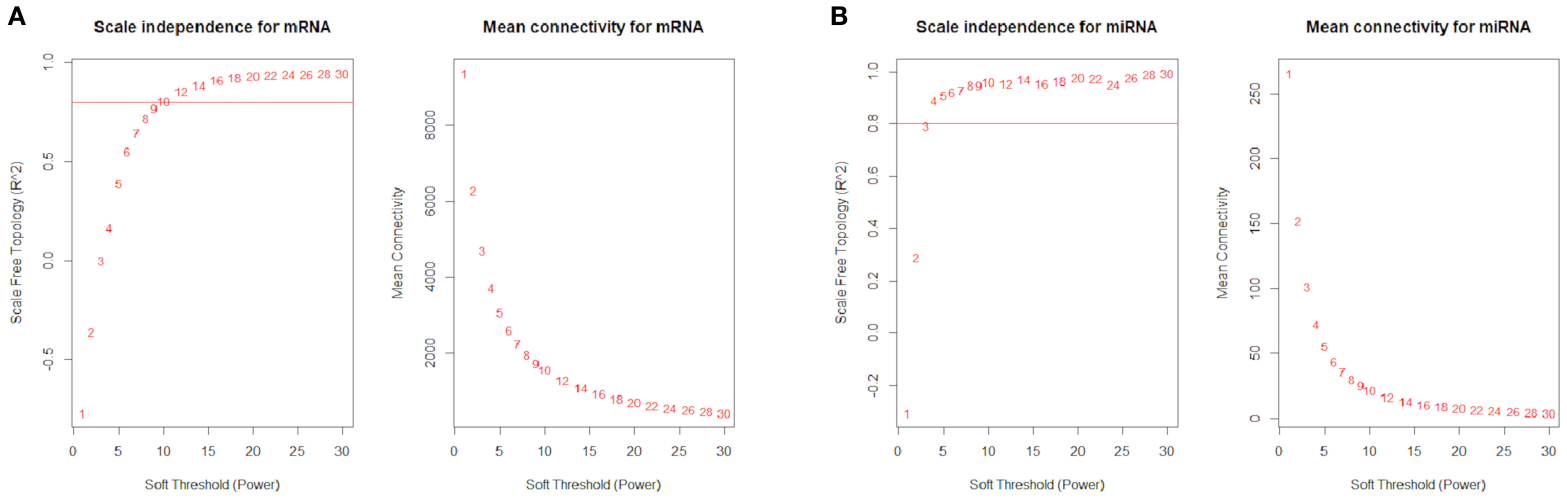
*R*^2^ of the free-scale topology and mean connectivity with soft threshold (power) for **(A)** mRNA data and **(B)** miRNA data.

The R package WGCNA (37) was used to construct the co-expression network. It constructs a similarity matrix by Pearson correlation coefficients to measure the similarity between the gene expression profiles and then transforms the similarity matrix into an adjacency matrix (A) raised to a β exponent (soft threshold) based on the free-scale topology model. In this study, a total of 18,329 genes were filtered from 26,332 genes in mRNA data based on the median absolute deviation (MAD) of each gene bigger than 0.01. The β power parameter (soft threshold) was equal to 12 when the *R*^2^ of the free-scale topology was equal to 0.8 (Figure 2A). In the miRNA data, 650 miRNAs were filtered from 765 genes, and the β power parameter (soft threshold) was equal to 4 (Figure 2B).

We chose the soft threshold power (β = 12 for mRNA and β = 4 for miRNA) based on the criterion of approximate scale-free topology to construct a weighted gene network and detect the consensus modules with the topological overlap matrix (TOM). The minimum module size was set at 30 for both miRNA data and mRNA data, and the maximum module sizes were set at 18,329 and 650 for mRNA data and miRNA data, respectively. Based on the dissimilarity between module eigengenes (MEs), the modules can be merged, where the first principal component of each module represents the gene expression profiles within the modules (38). Here, we set the cut height for module merging at 0.25, so the modules whose eigengenes are correlated above 0.75 will be merged.

A module association analysis was conducted between the ME and the RSV treatment status (i.e., 0, 0, 0, 1, 1, 1 for three control and three case groups, respectively) to calculate the correlations for the relevant module identifications. We calculated the module significance (MS) [i.e., the average absolute gene significance (GS) of all the genes involved in the module, where GS is measured as log *P-*value in the linear regression between gene expression and RSV treatment status] to evaluate the correlation strengths. Normally, the module with the highest MS score is the key module (37). Module significance genes in the association analysis (*P*-value < 0.1) were assigned for functional enrichment analysis. The hub genes were defined as the TOM values up to 0.8.

### Gene Ontology and Pathway Enrichment Analysis

R package *clusterProfiler* (version 3.6) (39) was used to test the Gene Ontology (GO) terms and Kyoto Encyclopedia of Genes and Genomes (KEGG) pathway enrichments. Significant enrichment was defined with the adjusted *P*-value < 0.1 for both GO terms and pathways.

### Predicted Target mRNAs of miRNA and mRNA–miRNA Networks

The bovine genomic sequence (release-99) and the gene annotation file were downloaded from the Ensembl FTP site (http://www.ensembl.org/index.html). We used TBtools to obtain the 3′ UTR sequence of the bovine genomic transcripts (40). Then, the transcript stable IDs were converted to the Ensembl stable IDs using the BioMart website (http://www.ensembl.org/biomart). Accordingly, we found the 3′ UTR sequence of the genes in the turquoise modules. The binding capability of miRNAs and their target genes in the turquoise modules was assessed by RNAhybrid (version 2.1.2) (41), with the minimal free energy hybridization under −20 and the helix constraint from 8 to 12.

## Results

### Differentially Expressed mRNAs and miRNAs Between the RSV Treatment and Control Groups

From our previous study (31, 32), a total of 3,856 DE mRNAs were identified from 18,329 mRNAs based on the threshold of adjusted *P*-value < 0.05; meanwhile, 93 DE miRNAs were also identified from 650 miRNAs based on the same thresholds (Table 1). The details of log_2_FC, *P*-value, adjusted *P*-value, and DE mRNAs with FPKM and miRNAs with TPM of each sample are listed in the Supplemental File 1.

**Table 1:** Summary of differentially expressed mRNAs and miRNAs.

	**DE mRNA (adjusted *P*-value <0.05)**	**DE mRNA (adjusted *P*-value <0.0001)**	**DE miRNA (adjusted *P*-value <0.05)**
Up-regulated	1,805	450	44
Down-regulated	2,051	681	49
Total	3,856	1,131	93

*Differentially expressed (DE) mRNA and miRNA results were achieved from our previous study (31, 32)*.

### Module Identification of the Gene Co-expression Network for mRNA and miRNA and Their Associations With RSV Treatment

**Figure 3:**
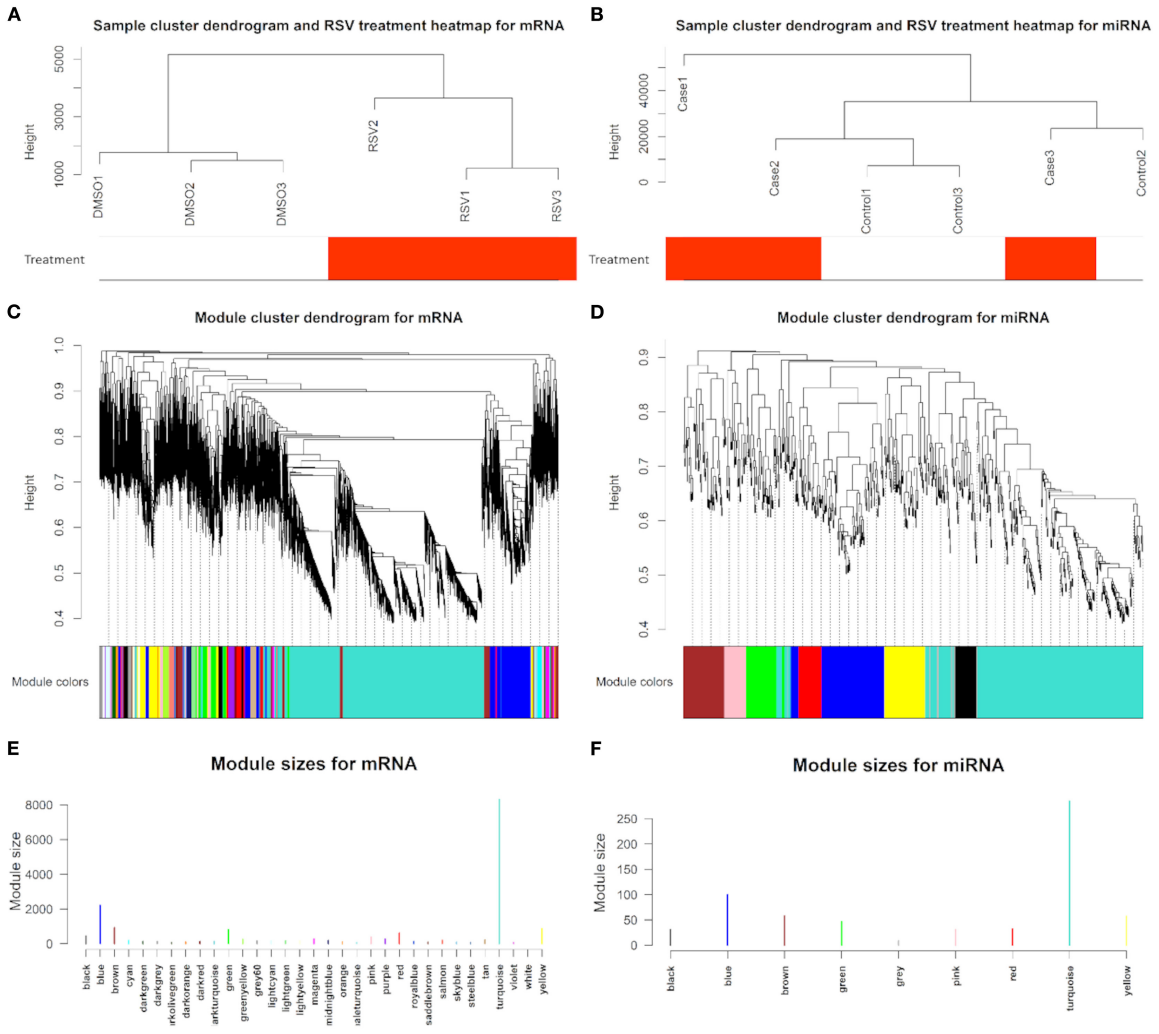
Sample cluster with RSV treatment heatmap, dendrogram clustering plots with assigned module colors based on topological overlap analysis of dissimilarity, and identified module size in different colors for **(A,C,E)** mRNAs and **(B,D,F)** miRNAs, respectively.

Using 18,329 mRNA and 650 miRNA data for the sample clustering, we found that the samples with RSV treatment were clustered together in mRNA analysis (Figure 3A), while RSV-treated samples were not clustered together by miRNA data (Figure 3B). Generally, 18,329 mRNAs were grouped into 32 modules that had similar co-expressions using the average linkage hierarchical clustering algorithm (Figures 3C,E), where 8,311 mRNAs were grouped into turquoise module as the key module, followed by 2,210 mRNAs into blue module, etc. (Figure 3E; Table 2). However, miRNAs were only grouped into eight modules (Figures 3D,F; Table 2), where 285 miRNAs were grouped into turquoise modules as the key module, followed by 100 miRNAs into blue modules. The mRNAs and miRNAs that were not assigned to any modules were grouped into gray modules (Figures 3C–F).

**Table 2:** Summary of mRNA and miRNA module identifications.

**Transcriptome sequencing**	**Modules**					
	**Turquoise**	**Blue**	**Brown**	**Yellow**	**Green**	**Red**
mRNAs (*n* = 18,329)	8,311 (45.34%)	2,210 (12.06%)	925 (5.05%)	886 (4.83%)	824 (4.50%)	613 (3.34%)
miRNAs (*n* = 650)	285 (43.85%)	100 (15.38%)	58 (8.92%)	57 (8.77%)	47 (7.23%)	32 (4.92%)

**Figure 4:**
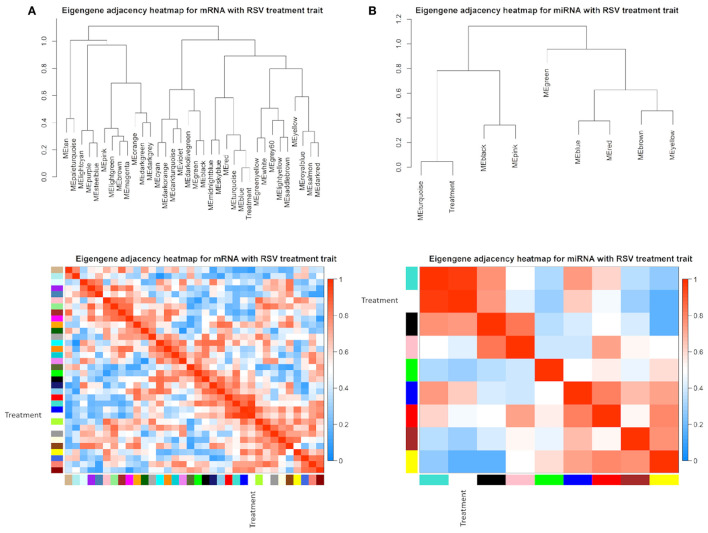
Eigengene dendrogram and adjacency heatmap of different co-expression modules for **(A)** mRNAs and **(B)** miRNAs, respectively.

The eigengene adjacency heatmap indicated that these modules of mRNAs and miRNAs could be clustered further together into groups (Figure 4). After incorporating the RSV treatment trait, we found that the treatment status was clustered with blue and turquoise modules for mRNAs (Figure 4A) and with turquoise modules in a single cluster for miRNAs (Figure 4B).

**Figure 5:**
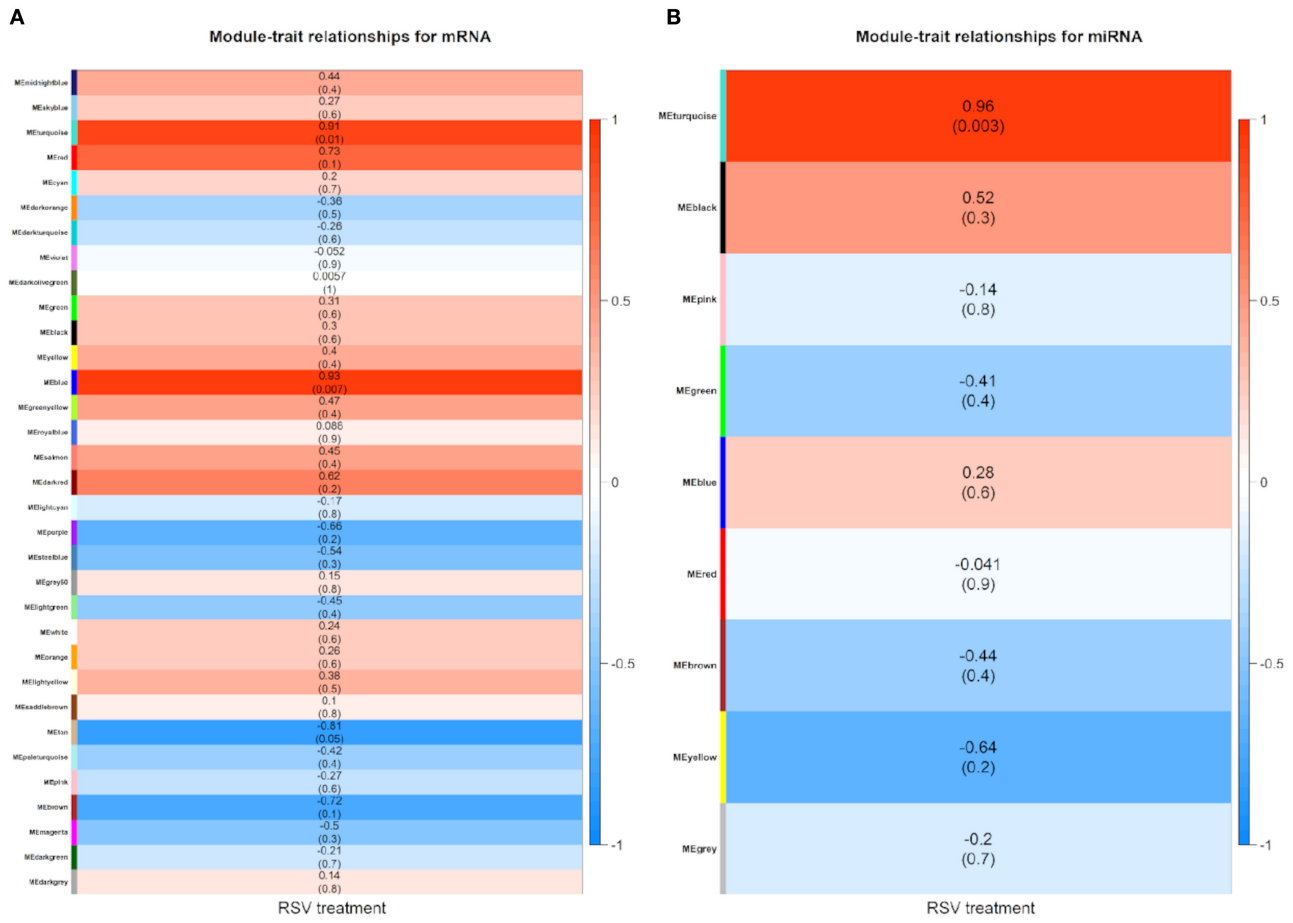
Module–trait relationship heatmap between RSV treatment and control groups for **(A)** mRNAs and **(B)** miRNAs, respectively. Each row indicates module eigengenes with the correlation coefficients (*P*-values in the brackets), where the red color represents positive correlation and the blue color represents negative correlation.

The module–trait relationship results revealed that RSV treatment had high correlations with turquoise module (0.91, *P*-value = 0.01), blue module (0.93, *P*-value < 0.01), and tan module (−0.81, *P*-value = 0.05) using mRNA data (Figure 5A), whereas only turquoise module (0.96, *P*-value < 0.01) was highly correlated with treatment status using miRNA data (Figure 5B). Therefore, the turquoise module showed a strongly positive relationship with RSV treatment no matter whether mRNA data or miRNA data is used.

**Figure 6:**
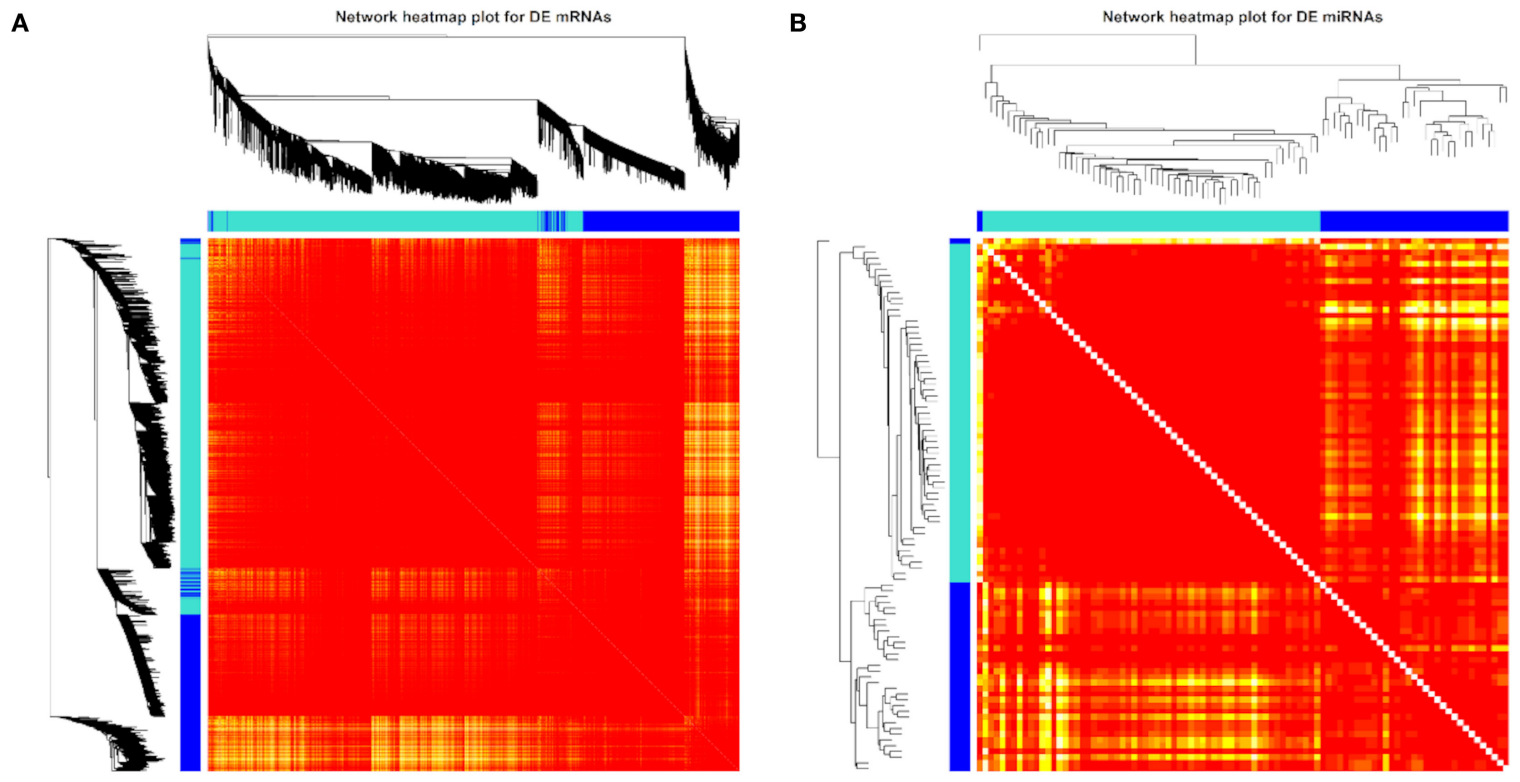
Weighted differentially expressed (DE) **(A)** mRNA (*n* = 3,856) and **(B)** miRNA (*n* = 93) network heatmap of the co-expression interactions based on the topological overlap matrix (TOM) dissimilarity. The gene dendrogram and module assignment are shown along the left side and the top, where the axe colors indicate the different modules. The color intensity inside the heatmap represents the overlap degree, where a light color represents low overlap and a darker red color represents higher overlap. DE mRNA and miRNA results were achieved from our previous study (31, 32).

We also used the 3,856 DE mRNAs and 93 DE miRNAs for the weighted gene network construction based on the same soft threshold power (β = 12 for mRNA and β = 4 for miRNA) and then visualized them in the TOM clusters (Figure 6). Here, the minimum module size and maximum block size were set at 30 and 3,856, respectively, for mRNA and were set at 30 and 93, respectively, for miRNA. Only two cluster modules were displayed for both mRNAs and miRNAs, i.e., turquoise and blue modules. In the turquoise module, 2,579 DE mRNAs and 59 DE miRNAs were found, while 1,277 DE mRNAs and 34 DE miRNAs were found in the blue module. Furthermore, the network heatmap of DE mRNAs and DE miRNAs showed a high level of overlap densities among the two clusters (Figure 6).

### Gene Ontology and Pathway Enrichment Analysis

**Figure 7:**
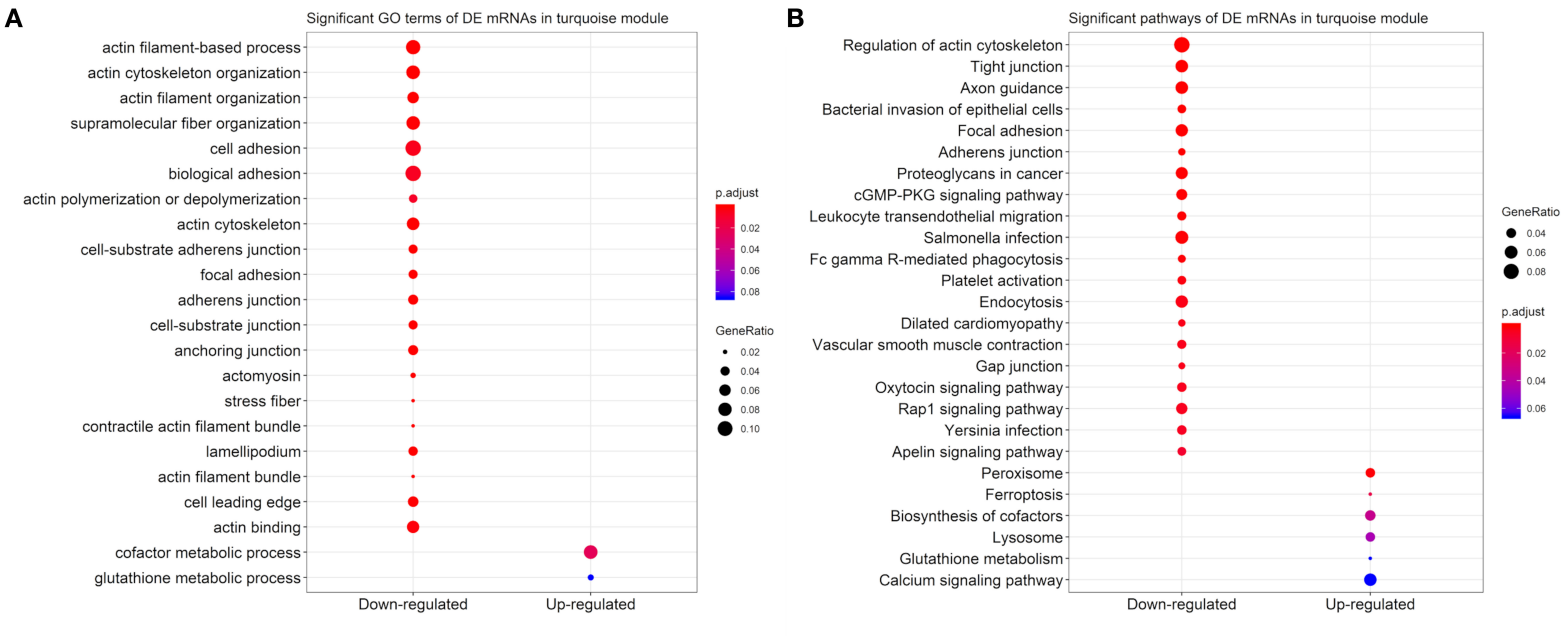
Scatter plots for the significant **(A)** Gene Ontology (GO) terms and **(B)** Kyoto Encyclopedia of Genes and Genomes (KEGG) pathways in the down-regulated and up-regulated categories of 2,579 differentially expressed (DE) mRNAs in turquoise module. Only the top 20 significant GO terms and KEGG pathways were visualized.

Based on the 2,579 DE mRNAs in the turquoise module, we performed an enrichment analysis to reveal significant GO terms and KEGG pathways. We found that the three most significant GO terms were actin filament-based process (GO:0030029, adjusted *P*-value = 1.86 × 10^−6^) with 37 DE genes that were enriched in, followed by actin cytoskeleton organization (GO:0030036, adjusted *P*-value = 4.33 × 10^−6^) with 34 DE genes, and actin filament organization (GO:0007015, adjusted *P*-value = 6.86 × 10^−5^) with 24 DE genes in the down-regulated category (Figure 7A). Similarly, the three most significant pathways were regulation of actin cytoskeleton (bta04810, adjusted *P*-value = 1.40 × 10^−11^) with 47 genes, tight junction (bta04530, adjusted *P*-value = 2.32 × 10^−6^) with 33 genes, and axon guidance (bta04360, adjusted *P*-value = 2.32 × 10^−6^) with 33 genes (Figure 7B).

### Networks Displaying the Relationships Among Genes Within Co-expressed Modules

**Figure 8:**
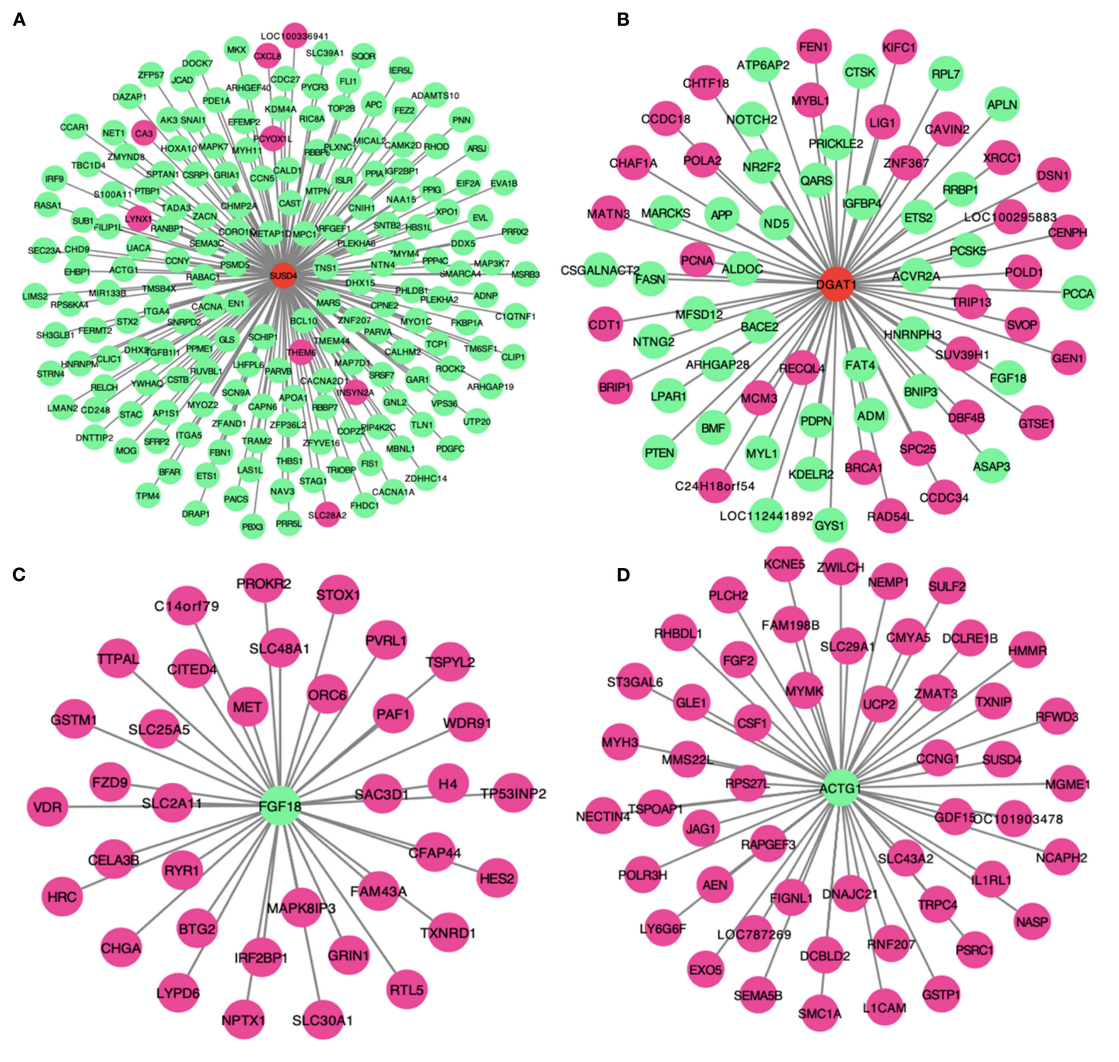
The networks displaying the relationships of four key hub genes within co-expressed turquoise modules, i.e., **(A)** sushi domain containing 4 (*SUSD4*) gene, **(B)** diacylglycerol O-acyltransferase 1 (*DGAT1*) gene, **(C)** fibroblast growth factor 18 (*FGF18*) gene, and **(D)** actin gamma 1 (*ACTG1*) gene.

We selected and constructed the network of four genes within the turquoise module including two significantly up-regulated genes, i.e., sushi domain containing 4 (*SUSD4*) gene and diacylglycerol O-acyltransferase 1 (*DGAT1*) gene, and two significantly down-regulated genes, i.e., fibroblast growth factor 18 (*FGF18*) gene and actin gamma 1 (*ACTG1*) gene (Figure 8). *SUSD4* has associations with 186 genes including 8 up-regulated genes and 176 down-regulated genes (Figure 8A). *DGAT1* was closely connected with 70 genes where 32 genes were up-regulated (e.g., proliferating cell nuclear antigen, *PCNA*) and 38 genes were down-regulated (e.g., phosphatase and tensin homolog, *PTEN*) (Figure 8B). *FGF18* and *ACTG1* were negatively connected with 36 and 51 up-regulated genes, respectively (Figures 8C,D). The selected hub gene *ACTG1* was negatively related with skeletal muscle myosin heavy chain 3 (*MYH3*) gene, fibroblast growth factor 2 (*FGF2*) gene, and uncoupling protein-2 (*UCP2*) (Figure 8D).

### Identification of miRNAs in the Key Module and Target Gene Prediction of the miRNAs for mRNA–miRNA Network

**Figure 9:**
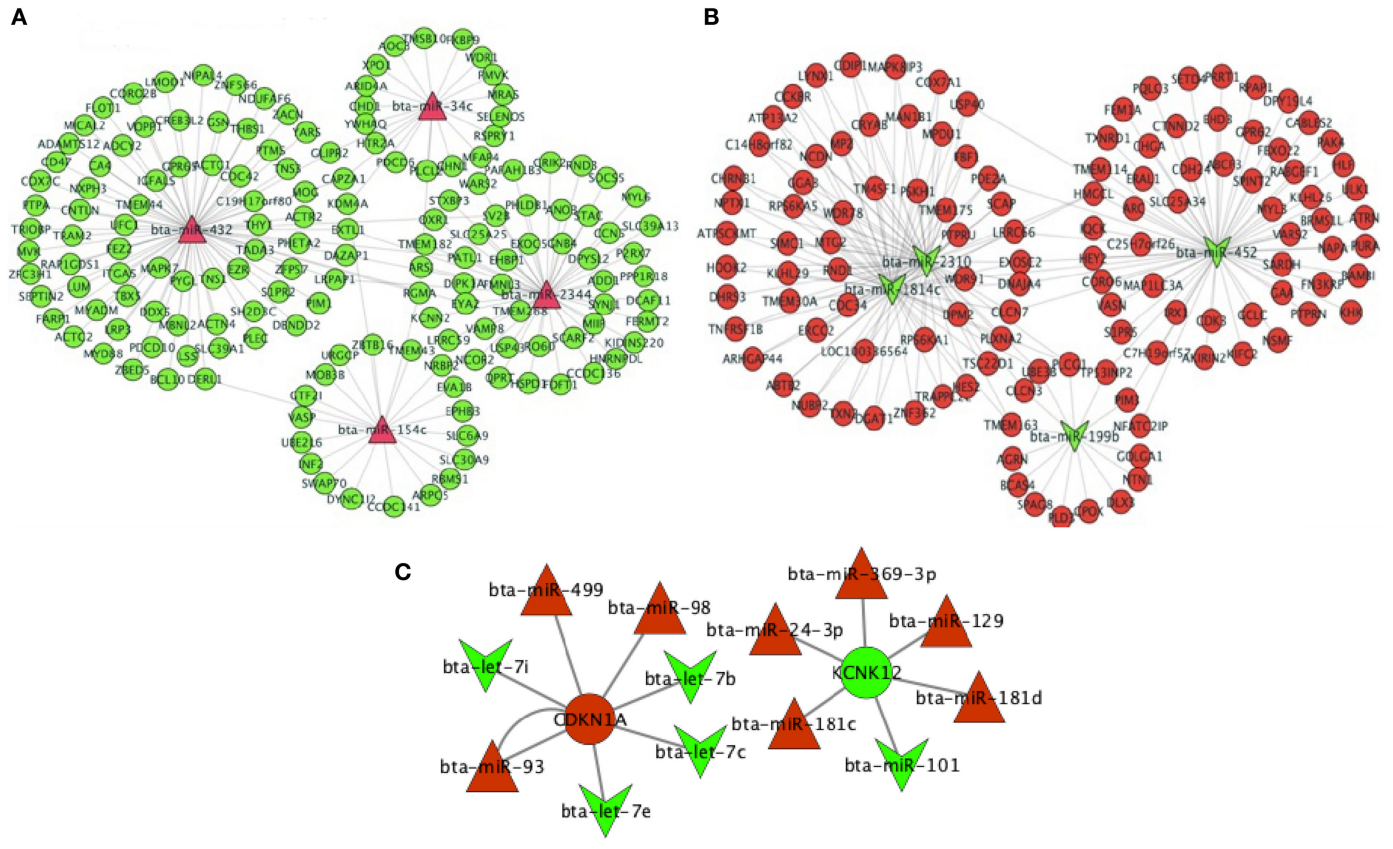
mRNA–miRNA network of the top four differentially expressed (DE) miRNAs in the **(A)** up-regulated status (bta-miR-34c, bta-miR-432, bta-miR-2344, and bta-miR-154c) and the **(B)** down-regulated status (bta-miR-199b, bta-miR-2310, bta-miR-1814c, and bta-miR-452) in the key turquoise module and their targeted DE genes. **(C)** mRNA–miRNA network of the top two DE genes and the targeting DE miRNAs. DE mRNA and miRNA results were achieved from our previous study (31, 32). The green color indicates the down-regulated miRNAs and genes, while the red color indicates the up-regulated miRNAs and genes.

Based on the top significant RSV-induced up- and down-regulated DE miRNAs in the turquoise module, we predicted their target genes. The top four up-regulated miRNAs were bta-miR-34c, bta-miR-432, bta-miR-2344, and bta-miR-154c that targeted 21, 78, 22, and 49 down-regulated genes in the turquoise module, respectively (Figure 9A). Likewise, the top four down-regulated miRNAs, i.e., bta-miR-2310, bta-miR-452, bta-miR-1814c, and bta-miR-199b targeted 59, 62, 58, and 15 up-regulated genes in the turquoise module, respectively, where bta-miR-2310 and bta-miR-1814c targeted the same genes (*n* = 57) (Figure 9B). Three up-regulated and three down-regulated miRNAs targeted the top up-regulated *CDKN1A* [adjusted *P*-value = 2.50 × 10^−104^ and log_2_(FC) = 1.97], while five up-regulated and one down-regulated miRNAs targeted the top down-regulated *KCNK12* [adjusted *P*-value = 5.92 × 10^−63^ and log_2_(FC) = −2.32] (Figure 9C).

## Discussion

Carcass weight is mainly influenced by the number and the size of myoblasts that are generated from somite and through proliferation, differentiation, and fusion into myofibers in an embryonic stage (42). Skeletal muscle fiber characteristics can be divided into fast and slow types based on the contraction speed that can determine the meat quality traits such as marbling (43). Internal (heredity) and external factors (nutrition and environment) are combined to regulate the conversions among the fiber types, such as arginine (44) and linoleic acid (45) that have been considered as the nutrients that influence the conversion of skeletal fiber type. Researchers also analyzed the omics data to reveal the effects of functional feed additives to improve carcass characteristics and beef quality, such as vitamin A, zinc propionate, etc. (46, 47). Our study aims to illustrate the additive effects of natural polyphenol compound RSV on primary bovine myoblast differentiation through transcriptome sequencing.

Resveratrol effects have been extensively studied on various cell types including cardiomyoblasts (8), fibroblasts (7), hepatocytes (48), smooth muscle cells (49), mammary epithelial cells (50), immune cells (6), and a multitude of various cancer cell lines (3). Moreover, researchers paid more attention to the RSV functions on human myoblast proliferation, injury, and death, as well as the resistance against oxidative stress of RSV to the skeletal muscle tissues in livestock (4, 11, 17, 20, 31). In this study, we identified biologically relevant key modules of the co-expressed mRNA and miRNA networks for the differentiation of bovine primary myoblast after RSV treatment. The results showed that the significant modules of DE mRNAs and DE miRNAs were strongly associated with RSV treatment status; for example, the key mRNA module was turquoise module with 2,579 DE mRNAs induced by RSV, where 47 down-regulated DE genes were enriched in the regulation of actin cytoskeleton pathway (Figure 7B). The actin cytoskeleton is essential for cell proliferation, differentiation, migration, phagocytosis, and exocytosis, even when cells experience oxidative damage (51). The actin cytoskeleton is also essential to maintaining the stability of skeletal muscle functions. The absence of *ACTG1* resulted in muscle weakness and a progressive myopathy in mice (52); meanwhile, the reduced expression of *ACTG1* was closely associated with up-regulated *MYH3* induced by RSV (Figure 8D). *MYH3* plays an important role in the skeletal muscle metabolism and the content of distinct types of skeletal muscle fibers (53). Thus, we suggested that RSV had functions on the type switch of the primary bovine myoblast fiber, which was consistent with the previous studies of RSV effects on C2C12 cells (54, 55).

Skeletal muscle development is elaborately regulated by myogenic regulatory factors (MRFs), growth factors (e.g., TGF-β and IGFs), signal pathways (e.g., IGF1-Akt-mTOR and Smad2/3 pathway), and non-coding RNAs (miRNAs) (56–58). MiRNAs play important roles in regulating myogenesis and regeneration, hypertrophy and atrophy, muscle disease, and aging (59, 60). In recent years, the identified functions of miRNAs in skeletal muscle development have been widely studied in cattle. Our study also identified 59 DE miRNAs in the turquoise module including bta-miR-432 and bta-miR-365-3p. They were highly expressed in skeletal muscle tissues and differently expressed in the fetal and adult stages of Qinchuan cattle; they may participate in the myoblast differentiation with vital roles (61, 62).

Network construction that integrates miRNA and mRNA data to identify the complex transcriptional regulating mechanism of RSV is more significant to the biological pathways for primary bovine myoblast than a separate analysis. This study found that *ACTG1* could be targeted by the significantly RSV-induced up-regulated bta-miR-432 that potentially activated the IGF2/AKT signaling pathway to promote the proliferation and differentiation of myoblasts (61) (Figure 9A). Interestingly, the RSV-induced down-regulated bta-miR-2310 and bta-miR-1814c could co-target 57 RSV-induced up-regulated DE genes, such as *DGAT1* that is the functional candidate gene for the improvement of meat and carcass fatness quality in beef cattle (63) (Figure 9B). *DGAT1* was also positively connected with the myoblast proliferation gene (*PCNA*) and negatively related with *PTEN*, which regulated the skeletal satellite cell proliferation and differentiation (64) (Figure 9B). The RSV-induced miRNAs participated in the complex co-expression networks to regulate primary myoblast differentiation through affecting mRNA expressions, which subsequently participated in regulating skeletal development, metabolism, and skeletal muscle fiber type switch, thereby functionally regulating the cattle carcass weight and meat quality.

## Conclusions

In summary, RSV treatments had high correlations with the turquoise module (0.91, *P*-value = 0.01) and blue module (0.93, *P*-value < 0.01) using mRNA data, but only had high correlations with the turquoise module (0.96, *P*-value < 0.01) using miRNA data. The two top GO terms of actin filament-based process (GO:0030029) and actin cytoskeleton organization (GO:0030036) and the two top KEGG pathways of regulation of actin cytoskeleton (bta04810) and tight junction (bta04530) were revealed using 2,579 DE genes in the turquoise module. The mRNA–miRNA network was then constructed based on the co-expressions of DE mRNA and miRNA in the key module. Our study provided a better understanding of the roles of RSV in inducing miRNA and of the characteristics of DE miRNAs in the key co-expressed module in regulation of mRNAs and revealed new candidate regulatory miRNAs and genes for beef quality traits.

## Data Availability Statement

The datasets presented in this study can be found in online repositories. The names of the repository/repositories and accession number(s) can be found below: GEO, GSE186730.

## Ethics Statement

All the animal procedures were carried out according to the protocols approved by the College of Animal Science and Technology, Northwest A&F University, China. All the experimental animals were approved by the Institutional Animal Care and Use Committee in the College of Animal Science and Technology, Northwest A&F University, China.

## Author Contributions

DH performed all the experiments. DH and XW analyzed the data and wrote the manuscript. XW, YY, BT, and L-EH improved the manuscript. HC and BH conceived and designed the experiments. KQ collected experimental samples. All authors contributed to the article and approved the submitted version.

## Funding

This work was supported by the National Natural Science Foundation of China (No. 31772574), the Program of National Beef Cattle and Yak Industrial Technology System (CARS-37), the Program of Yunling Scholar and the Young and Middle-aged Academic Technology Leader Backup Talent Cultivation Program in Yunnan Province, China (No. 2018HB045), the Yunnan Provincial Major S&T Project (2019ZG007 and 2019ZG011), and the Doctoral Startup Project of Chuxiong Normal University (No. BSQD2101).

## Conflict of Interest

XW was employed by company Konge Larsen ApS. The remaining authors declare that the research was conducted in the absence of any commercial or financial relationships that could be construed as a potential conflict of interest.

## Publisher's Note

All claims expressed in this article are solely those of the authors and do not necessarily represent those of their affiliated organizations, or those of the publisher, the editors and the reviewers. Any product that may be evaluated in this article, or claim that may be made by its manufacturer, is not guaranteed or endorsed by the publisher.

## Abbreviations

*ACTG1*: actin gamma 1 gene
*DGAT1*: diacylglycerol acyltransferase-1 gene
DE: differentially expressed
FPKM: fragments per kilobase of transcript sequence per million base pairs sequenced
FDR: false discovery rate
FCs: fold changes
GO: Gene Ontology
IACUC: Institutional Animal Care and Use Committee
MAD: median absolute deviation
mRNAs: messenger RNAs
MRFs: myogenic regulatory factors
*MEF2*: myocyte enhancer factor 2
RSV: resveratrol
TPM: transcript per million
KEGG: Kyoto Encyclopedia of Genes and Genomes
WGCNA: weighted gene co-expression network analysis.
